# Supplementary material for: Ortholog Analysis and Transformation of Glycoside Hydrolase Genes in Hyperthermophilic Archaeal Thermococcus Species
Source: Int J Mol Sci. 2025 Apr 2;26(7):3305. doi: 10.3390/ijms26073305 (PMC11989552; doi:10.3390/ijms26073305)
Supplement: Supplementary file 1 [file ijms-26-03305-s001.zip › ijms-3514586-supplementary.pdf]

Supplementary Table S1. The list of strains and fosmids used in this study.

Strains/Fosmids	Description	Reference
**Strains**		
E. coli		
EPI300^{TM}-T1^{R}	Fosmid cloning host	EPICENTRE
T. onnurineus		
NA1	Wild-type strain	[35]
GH03	NA1 derivative pPAC-GHC1115 transformation, P_{0157}*hmg*$_{pfu}$-*Tpa*-GH gene cluster	This study
**Fosmids**		
pNA1comFosC1096	pCC1FOS carrying P_{0157} promotor, HMG cassette, and 1kbp Left-arm (LA) and Right-arm (RA) for homologous recombination of *T. onnurineus* NA1 genome; backbone fosmid for mutant construction; Sim^{r}	[42]
pTpaGH1	pNA1comFosC1096 carrying *Tpa*-GH region I gene cluster from *T. pacificus*	
pPAC-GHC1115	pNA1comFosC1096 carrying *Tpa*-GH gene cluster from *T. pacificus*	This study

Supplementary Table S2. PCR primers used in this study. Underlined nucleotides indicate restriction enzyme recognition sites.

**PCR region**	**Nucleotide sequence (5' to 3')**	
	**Forward**	**Reverse**
***Tpa*-GH region I**	GCTCATAAAATGCTTGGGAGATGACCTAGGTTAGTAAATTGACCTGAACAGCAC	TGAGGCTGGCCATCGTTGACGCCACGCATGACGCGTCTCTATTTATTGTCC
***Tpa*-GH region II**	CATTGGGACTTCAAGGGCATTCCCCACTATCCAACAGGAGGACAATAAATAG	CCATCGTTGACGCCACGCATGCTACCTCCTCACGAGGAAGTCTAAGCTGGA
